# The Hidden Burden: Impact of Allostatic Load on Colorectal Cancer Surgery Outcomes

**DOI:** 10.1245/s10434-025-17711-0

**Published:** 2025-06-21

**Authors:** Mujtaba Khalil, Selamawit Woldesenbet, Zayed Rashid, Abdullah Altaf, Shahzaib Zindani, Emily Huang, Syed Husain, Matthew Kalady, Samilia Obeng-Gyasi, Timothy M. Pawlik

**Affiliations:** Department of Surgery, The Urban Meyer III and Shelley Meyer Chair for Cancer Research, James Comprehensive Cancer Center, Wexner Medical Center, The Ohio State University, Columbus, OH USA

**Keywords:** Allostatic load, Surgical outcomes, Social vulnerability, Colorectal cancer

## Abstract

**Background:**

Allostatic load (AL) is a composite measure of the physiological damage caused by socioenvironmental stressors. We sought to investigate the association between AL, social vulnerability index (SVI), and postoperative outcomes following colorectal cancer (CRC) surgery.

**Patients and Methods:**

Individuals who underwent surgery for CRC between 2022 and 2024 were identified using the Epic Cosmos database. AL is calculated on the basis of ten biomarkers from four physiological systems (cardiovascular, metabolic, renal, immune). Multivariable regression models were utilized to examine the association between AL and postoperative outcomes.

**Results:**

Among 40,520 individuals, mean patient age was 67.7 years (SD ±13.9), roughly half of the patients were male (*n* = 20,573; 50.8%), and patients generally had a high Charlson comorbidity index score (CCI > 2; *n* = 33,132; 81.8%). Overall, 7.1% (*n* = 2897) of patients had a high AL. Notably, AL increased with increasing SVI (ref: low; medium: 1.10 [95% CI 1.01–1.20]; high: 1.17 [95% CI 1.07–1.28]). High AL was associated with a 48% increased risk of postoperative complications (OR 1.48; 95% CI 1.38–1.58), a 79% increased risk of an extended length of stay (OR 1.79; 95% CI 1.67–1.90), and a twofold (OR 2.13; 95% CI 1.90–2.37) increase in the risk of mortality within 30 days of surgery.

**Conclusions:**

Individuals with CRC living in socially vulnerable neighborhoods experience high physiological damage and are at a higher risk of postoperative complications and mortality. Therefore, patients from socially vulnerable neighborhoods may require preoperative screening and optimization to mitigate disparities in surgical outcomes.

**Supplementary Information:**

The online version contains supplementary material available at 10.1245/s10434-025-17711-0.

Recent advancements in cancer care have improved survival rates and have resulted in a better quality of life.^1^ However, these benefits have not been experienced equally across different racial and socioeconomic groups.^2,3^ In particular, individuals residing in socially vulnerable neighborhoods experience significant economic stressors, including unemployment, poverty, food insecurity, and debt.^4^ Moreover, these individuals encounter social challenges such as limited access to transportation and green spaces and increased exposure to environmental hazards.^5^ Chronic psychological and physiological stress can lead to epigenetic changes that disrupt biological pathways, ultimately resulting in worse health outcomes.^6,7^

When experiencing physiologic stress, environmental changes, or challenges, the body undergoes allostasis.^7^ Allostasis refers to the body’s adaptive mechanisms that adjust and recalibrate physiological systems to maintain balance and overall health.^8^ However, when environmental challenges exceed an individual’s ability to cope, allostatic overload occurs.^9^ This results in a heightened state in which stress response systems are repeatedly activated, buffering mechanisms fail, and multiple physiological systems become disrupted.^6^ Of note, allostatic load (AL) is measured using vital signs, anthropometric measurements, and routine laboratory assessments.^10^ These indicators reflect derangements in multiple physiological systems, including metabolic, immune, cardiac, and hematologic functions.^6^ Previous studies have demonstrated an association between high AL scores and various adverse health outcomes.^11,12^ For instance, Carbone et al. observed that elevated AL was linked to cognitive and memory decline.^11^ Similarly, Stabellini et al. reported that high AL scores increased the risk of peripheral vascular disease and other cardiovascular conditions.^12^

Prior research has highlighted a strong association between living in a socially vulnerable neighborhood and suboptimal surgical outcomes.^13^ These poor outcomes maybe due to the physiological damage caused by chronic exposure to psychological and physical stressors.^10^ Nonetheless, the association between social vulnerability, AL, and surgical outcomes following cancer surgery remains poorly defined. Therefore, the objective of the current study was to determine the association between social vulnerability, AL, and postoperative complications among patients with colorectal cancer (CRC). We hypothesized that high AL among individuals living in socially vulnerable neighborhoods may be associated with poor postoperative outcomes.

## Patients and Methods

### Data Source and Patient Selection

The Epic Cosmos database was queried using International Classification of Diseases Tenth Edition (ICD-10) codes to obtain data on individuals who underwent CRC surgery between 2013 and 2020. Epic Cosmos database aggregates deidentified electronic health record (EHR) data from participating health systems that use Epic as their EMR platform across the USA.^14^ Not all health systems that use Epic contribute data; participation is voluntary and subject to institutional agreements.^14^ Of note, Epic Cosmos is one of the largest nationwide health systems databases covering 257 million unique patients and more than 36,000 hospitals and clinics.^14^ One key strength of the Epic Cosmos database is the availability of socioeconomic data at the ZIP code level in addition to the clinical data.^15^ The study included individuals aged 18 years and older who had newly diagnosed CRC and underwent surgical treatment. Patients who did not undergo surgery, had stage 0 or IV disease, or had missing outcome data were excluded. This study followed the Strengthening the Reporting of Observational Studies in Epidemiology (STROBE) reporting guideline for observational studies. The Institutional Review Board at Ohio State University approved this study, and informed consent was waived because the data was deidentified.

### Allostatic Load

**Fig. 1:**
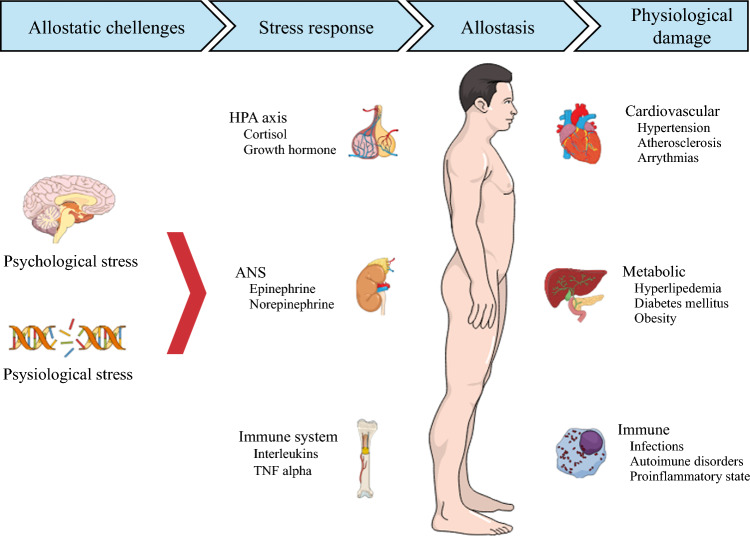
Schematic representation of allostasis and associated physiological damage

AL is a composite measure of the physiological damage caused by cognitive and emotional stressors (Fig. 1). AL can be calculated using a variety of biomarkers that reflect physiologic dysregulation across multiple systems.^10,16^ Ten biomarkers were selected on the basis of both prior evidence and practical availability within the Epic Cosmos database, in alignment with previously validated methodologies.^10,16,17^ The key strength of the allostatic load construct is its capacity to aggregate data from multiple systems, thus reducing the emphasis on any single biomarker.^10^ Specifically, laboratory test results from the year prior to CRC surgery were screened, and ten biomarkers from four distinct physiological systems were included.^10^ The four physiological systems and their respective biomarkers were: (1) cardiovascular—heart rate, systolic blood pressure, and diastolic blood pressure; (2) metabolic—body mass index, alkaline phosphatase, blood glucose, and albumin; (3) renal—creatinine and blood urea nitrogen; and (4) immunologic—white blood cell count.^10^ The distribution of each biomarker was assessed, and patients were assigned a point if their biomarker level fell within the worst quartile. For example, values ≥ 75th percentile for heart rate, blood pressure (systolic and diastolic), body mass index, alkaline phosphatase, blood glucose, white blood cell count, creatinine, and blood urea nitrogen were each assigned a point. Similarly, values ≤ 25th percentile for albumin were assigned a point. The assigned points were summed to create a composite AL score (range 0–10), with a higher score reflecting greater physiological damage. AL scores were then dichotomized into high and low categories, using the cohort’s median AL score as the cutoff.

### Covariates and Outcomes of Interest

Patient-level data on age, sex, race (categorized as white, Black, Asian, or other [Native American, Hispanic, and Alaska Native]), marital status, Charlson comorbidity index (CCI), cancer type (colon versus rectum), surgical approach (open versus minimally invasive), year of diagnosis, Social Vulnerability Index (SVI), and residential area (metropolitan versus nonmetropolitan) were collected. SVI is a composite measure developed by the Centers for Disease Control and Prevention (CDC), incorporating 18 variables across 4 domains: socioeconomic status, household composition and disability, minority status and language, and housing type and transportation.^18^ The SVI data are precalculated and available within the Epic Cosmos database.^14^

The primary outcomes of interest were postoperative complications, extended length of stay (LOS), and 30-day mortality. Extended LOS was defined as an inpatient stay greater than the 75th percentile. Postoperative complications were identified using ICD-10 diagnosis codes, as previously described.^19,20^ These complications included surgical site infection, pneumonia, myocardial infarction, venous thromboembolic event (deep vein thrombosis and pulmonary embolism), acute kidney injury, pulmonary failure, and hemorrhage. Discharge to home was studied as a secondary outcome.

### Statistical Analysis

Descriptive statistics were presented as median values with interquartile range (IQR) for continuous variables and frequency and percentage (%) for categorical variables. Differences in baseline characteristics were assessed using the Kruskal–Wallis test for continuous variables and either the chi-squared test or Fisher’s exact test for categorical variables. Multivariable logistic regression models were utilized to examine the association between SVI and AL, as well as between AL and postoperative complications. The models were adjusted for age, sex, marital status, CCI, year of diagnosis, procedure urgency, cancer site, surgical approach, and residential area. Missing covariates were addressed through the multiple imputation by chained equations (MICE) method.^21^ To evaluate the robustness of the results, the analyses were conducted on both the imputed and non-imputed datasets, and the outcomes were consistent. Statistical tests were conducted using a two-tailed approach with a significance level of *p* < 0.05. Analyses were performed using SAS 9.4 (SAS Institute).

## Results

### Baseline Characteristics of Patients

A total of 40,520 patients with CRC underwent surgery (colon: *n* = 32,366, 79.9%; rectum:* n* = 8154, 20.1%). Mean age at the time of surgery was 67.7 years (SD ±13.9), and majority of the patients had a high CCI score (CCI >2: *n* = 33,132, 81.8%). Most patients were male (*n* = 20,573, 50.8%), married (*n* = 21,216, 52.9%), and lived in metropolitan areas (*n* = 32,686, 81.4%), and a vast majority of the cohort self-identified as white (*n* = 31,951, 79.5%). Regarding surgical approach, 50.2% (*n* = 20,334) underwent open surgical procedure, while 49.8% (*n* = 20,186) underwent minimally invasive surgery (Table 1). Following surgery, 39.3% (*n* = 15,915) of patients experienced complications. These included myocardial infarction (*n* = 3024, 7.5%), venous thromboembolism (*n* = 1869, 4.6%), surgical site infection (*n* = 8775, 21.7%), acute kidney injury (*n* = 1060, 2.6%), pulmonary failure (*n* = 3171, 7.8%), and pneumonia (*n* = 1418, 3.5%). Moreover, 28.3% (*n* = 11,482) of patients had an extended LOS, 39.1% (*n* = 15,834) were discharged to a facility, and 5.4% (*n* = 2198) died within 30 days (Table 2).

**Table 1:** Baseline characteristics of patients stratified by high and low allostatic load

Characteristics	Total (*n* = 40,520)	Allostatic load		*p* value
		Low (*n* = 37,623, 92.9%)	High (*n* = 2897, 7.1%)	
Age	67.7 (13.9)	67.5 (14.0)	70.3 (12.5)	< 0.001
Sex				
Female	19,944 (49.2)	18,799 (50.0)	1145 (39.5)	< 0.001
Male	20,573 (50.8)	18,822 (50.0)	1751 (60.5)	
Marital status				
Single	18,860 (47.1)	17,436 (46.8)	1424 (49.9)	< 0.001
Married	21,216 (52.9)	19,785 (53.2)	1431 (50.1)	
Race				
White	31,951 (79.5)	29,847 (80.0)	2104 (73.2)	< 0.001
Black	5488 (13.7)	4891 (13.1)	597 (20.8)	
Asian	1249 (3.1)	1185 (3.2)	64 (2.2)	
Other	1478 (3.7)	1370 (3.7)	108 (3.8)	
Charlson comorbidity index				
≤ 2	7388 (18.2)	7144 (19.0)	244 (8.4)	< 0.001
> 2	33,132 (81.8)	30,479 (81.0)	2653 (91.6)	
Cancer site				
Colon	32,366 (79.9)	29,904 (79.5)	2462 (85.0)	< 0.001
Rectum	8154 (20.1)	7719 (20.5)	435 (15.0)	
Surgical approach				
Open	20,334 (50.2)	18,677 (49.6)	1657 (57.2)	< 0.001
Minimally invasive	20,186 (49.8)	18,946 (50.4)	1240 (42.8)	
Social vulnerability index				
Low	13,043 (32.6)	12,252 (33.0)	791 (27.7)	< 0.001
Medium	13,534 (33.8)	12,561 (33.8)	973 (34.0)	
High	13,463 (33.6)	12,367 (33.3)	1096 (38.3)	
Year of diagnosis				
2022	15,418 (38.1)	14,344 (38.1)	1074 (37.1)	0.433
2023	17,584 (43.4)	16,295 (43.3)	1289 (44.5)	
2024	7518 (18.6)	69,84 (18.6)	534 (18.4)	
Residential area				
Metropolitan	32,686 (81.4)	30,402 (81.6)	2284 (79.7)	0.015
Non-metropolitan	7459 (18.6)	6878 (18.4)	581 (20.3)

**Table 2:** Postoperative outcomes stratified by high and low allostatic load

Outcomes	Total (*n* = 40,520)	Allostatic load		*p* value
		Low (*n* = 37,623, 92.9%)	High (*n* = 2897, 7.1%)	
Extended length of stay	11,482 (28.3)	10,314 (27.4)	1168 (40.3)	< 0.001
30-day mortality	2198 (5.4)	1879 (5.0)	319 (11.0)	< 0.001
Complications	15,915 (39.3)	14,438 (38.4)	1477 (51.0)	< 0.001
Myocardial infarction	3024 (7.5)	2678 (7.1)	346 (11.9)	< 0.001
DVT and PE	1869 (4.6)	1683 (4.5)	186 (6.4)	< 0.001
Surgical site infection	8775 (21.7)	8100 (21.5)	675 (23.3)	0.026
Acute kidney injury	1060 (2.6)	899 (2.4)	161 (5.6)	< 0.001
Pulmonary failure	3171 (7.8)	2725 (7.2)	446 (15.4)	< 0.001
Pneumonia	1418 (3.5)	1223 (3.3)	195 (6.7)	< 0.001
GI hemorrhage	2165 (5.3)	1939 (5.2)	226 (7.8)	< 0.001
Discharge to home	24,686 (60.9)	23,253 (61.8)	1433 (49.5)	< 0.001

Median AL was 4 (IQR 2–7); 7.1% (*n* = 2897) of patients had high AL. Of note, older individuals (high AL: 70.3 years [SD ±12.5] versus low AL: 67.5 [SD ±14.0]) and patients with a high CCI (CCI > 2: high AL: 91.6% versus low AL: 81.0%) were more likely to have high AL (both *p* < 0.001). Similarly, Black patients (high AL: 20.8% vs. low AL: 13.1%) and individuals living in medium (high AL: 34.0% vs. low AL: 33.8%) or high (high AL: 38.3% versus low AL: 33.3%) SVI neighborhoods were also more likely to have high AL (all *p* < 0.001) (Table 2). In addition, older age (OR 1.01; 95% CI 1.02–1.02), male sex (OR 1.67; 95% CI 1.56–1.79), a high CCI score (OR 2.93; 95% CI 2.61–3.29), and residence in a non-metropolitan area (OR 1.10; 95% CI 1.01–1.20) were independently associated with high AL (Supplementary Table S1). Notably, the risk of high AL increased in a stepwise fashion with an increase in SVI (ref: low; medium: 1.10 [95% CI, 1.01–1.20]; high: 1.17 [95% CI, 1.07–1.28]).

### Allostatic Load and Postoperative Outcomes

**Fig. 2:**
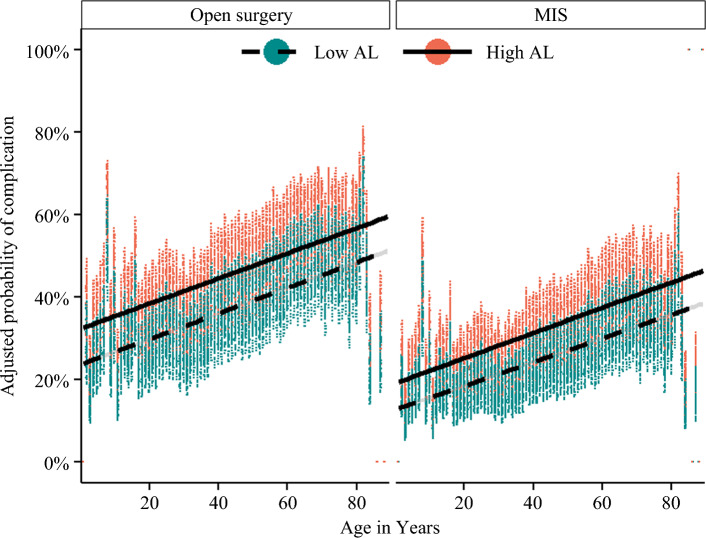
Adjusted risk of postoperative complications among patients with high and low allostatic load, stratified by type of surgical approach

Patients with high AL were more likely to experience postoperative complications (high AL: 51.0% versus low AL: 38.4%) and have an extended LOS (high AL: 40.3% versus low AL: 27.4%) (both *p* < 0.001). Specifically, patients with high AL were more likely to experience myocardial infarction (high AL: 11.9% versus low AL: 7.9%), venous thrombosis (high AL: 6.4% versus low AL: 4.5%), surgical site infection (high AL: 23.3% versus low AL: 21.5%), acute kidney injury (high AL: 5.6% versus low AL: 2.4%), and pulmonary failure (high AL: 15.4% versus low AL: 7.2%) (all *p* < 0.05). Moreover, individuals with high AL were less likely to be discharged to home (high AL: 49.5% versus low AL: 61.8%;* p* < 0.001) (Table 2). On multivariable regression analysis, high AL was associated with a 48% increased risk of any postoperative complication (OR 1.48; 95% CI 1.38–1.58), a 79% increased risk of an extended LOS (OR 1.79; 95% CI 1.67–1.90), and a twofold (OR 2.13; 95% CI 1.90–2.37) increase in the risk of mortality within 30 days of surgery (Table 3). These results remained consistent in sub-analysis based on CCI, surgical approach, and cancer site (Fig. 2 and Supplementary Table S2).

**Table 3:** Multivariable logistic regression examining the association between high allostatic load and postoperative outcomes (reference: low allostatic load)

Outcomes	Odds ratio	95% CI	*p* value
Postoperative complications	1.48	1.38–1.58	< 0.001
Extended length of stay	1.79	1.67–1.90	< 0.001
30-day mortality	2.13	1.90–2.37	< 0.001

## Discussion

There is a complex interplay between socioeconomic status and poor health, with previous studies highlighting social factors such as transportation, housing stability, and insurance as key mediators.^13^ While these social factors are undoubtedly important, there may also be a biological pathway in play.^16^ Individuals living in socially vulnerable neighborhoods are often exposed to chronic psychological and physiological stress.^20^ Stress activates allostasis, prompting the body to recalibrate its physiological systems to maintain balance and support overall health.^9^ However, prolonged or excessive activation of stress responses can disrupt biological pathways, leading to physiological damage.^9^ Nonetheless, the biological pathways linking socioeconomic stress to poor surgical outcomes remain poorly understood. Therefore, the current study was important because we leveraged national data from EPIC Cosmos, a novel, electronic medical-record-based database to characterize AL in socially vulnerable patients and examine its impact on surgical outcomes. Notably, the risk of high AL increased in a stepwise fashion with an increase in SVI. Additionally, high AL increased the risk of postoperative complications, 30-day mortality, and extended length of stay. These findings suggest that individuals from socially vulnerable neighborhoods experience poorer surgical outcomes due to elevated AL and disruptions in biological pathways.

When the body is exposed to stress, various physiological systems interact at different levels to adapt to the surrounding environment.^9^ For instance, the neuroendocrine system and hypothalamic-pituitary-adrenal axis respond to stressors by increasing the production of cortisol and catecholamines.^8^ This stress response involves the activation of the sympathetic nervous system, which triggers the release of epinephrine and norepinephrine, preparing the body for a fight-or-flight response.^22^ At the cellular level, selective gene activation occurs and the immune system responds by overproducing leukocytes, cytokines, and tumor necrosis factor.^23^ These adaptive responses are crucial for coping with immediate stress, but their prolonged activation results in disruption of multiple biological pathways and allostasis overload.^9^ The current study demonstrated similar biological responses, and the underlying physiological damage, as measured by AL, increased in a stepwise fashion with an increase in SVI. The changes in AL in response to socioeconomic stressors were consistent with previous studies. For example, Ribeiro et al. noted that individuals residing in areas with a high area deprivation index had higher levels of AL and lower levels of global DNA methylation.^24^ Similarly, Chen et al. demonstrated that lower scores on the Ohio Opportunity Index were associated with high AL.^16^ Specifically, individuals with low education, transportation challenges, and unstable housing experienced higher AL.^16^

While previous studies have highlighted a strong association between socioenvironmental stressors and postoperative outcomes, this is the first study to explore the relationship between the biological markers of internalized stress and postoperative outcomes in patients with CRC.^2,13^ Of note, patients with high AL have an increased risk of myocardial infarction, venous thrombosis, surgical site infection, acute kidney injury, pulmonary failure, pneumonia, and hemorrhage following CRC surgery. These findings are consistent with prior reports studying breast and lung cancer. For example, Obeng-Gyasi et al. noted that AL increases the risk of breast cancer, and following treatment, patients with high AL had a higher risk of all-cause mortality.^10^ Another study from the same group demonstrated that patients with high AL had a twofold higher risk of mortality following lung cancer surgery.^17^ Of note, patients with high AL experience poor outcomes due to prolonged activation of stress responses and dysfunction across the cardiovascular, immune, and metabolic systems.^23^ For example, elevated catecholamines result in hypertension and tachycardia, which increase the risk of stroke and myocardial infarction.^12^ Furthermore, a compromised immune system increases susceptibility to infections, autoimmune disorders, and delayed healing.^25^ This delay is further exacerbated by impaired cellular repair mechanisms, increased oxidative stress, and accelerated cellular aging secondary to chronic physiologic stress.^26^

The findings of the current study have several important clinical implications to improve surgical outcomes.^25,27^ AL is intended to complement, not replace, existing preoperative risk assessment protocols.^16^ Traditional tools such as the ASA physical status classification and CCI primarily focus on overt comorbidities, functional status, nutritional state, and lifestyle factors.^10^ In contrast, AL provides an integrated and biologically based measure of subclinical physiologic dysregulation across multiple organ systems, reflecting the cumulative burden of chronic stress that may not be captured through routine clinical assessment.^27^ Incorporating AL into clinical workflows may help enhance risk stratification and identify patients who could benefit from targeted prehabilitation or intensified perioperative optimization.^27^ Importantly, clinical diagnoses require significant physiological dysfunction to manifest, representing the “end product” of maladaptive adaptation.^28,29^ AL, by comparison, offers an early warning signal by capturing biochemical and neuroendocrine alterations that precede clinical disease.^25^ Another advantage of AL is its ease of use, as it is calculated from common biomarkers such as blood pressure, glucose levels, and inflammatory markers that are already routinely measured in preoperative care.^10^ Therefore, AL can be readily integrated into clinical practice without the need for costly or invasive testing, minimizing additional patient burden.^27^

Despite several strengths, including a large, nationally representative patient cohort, the findings of the current study should be interpreted with consideration of a few limitations. The cross-sectional design limits the ability to draw causal inferences. Moreover, the Epic Cosmos database does not capture detailed information on center-specific perioperative practices, such as bowel preparation protocols or implementation of enhanced recovery after surgery (ERAS) pathways. As such, unmeasured institutional variation may have contributed to differences in postoperative outcomes. Furthermore, the study relied on a single measurement of AL, preventing an assessment of fluctuations over time or the investigation of potential longitudinal effects. The study population was restricted to patients aged 18 years and older with CRC, limiting the generalizability of the findings to other age groups or different clinical diagnoses. Moreover, laboratory values used to calculate allostatic load were obtained within the 12 months preceding surgery. In cases where only preoperative values were available, these were included, which may reflect both acute disease-related physiological changes and chronic stress burden. Future studies utilizing serial or longitudinal biomarker measurements are needed to better distinguish chronic physiological stress from acute effects of cancer.

In conclusion, living in socially vulnerable areas exposes individuals to psychological and physiological stressors, which, over time, can lead to physiological damage. This underlying damage and disruption of biological pathways increase the risk of postoperative complications and mortality. As such, patients from socially vulnerable neighborhoods may require preoperative screening and optimization to mitigate disparities in surgical outcomes.

## Supplementary Information

Below is the link to the electronic supplementary material.

Supplementary file1 (DOCX 17 KB)

## Data Availability

The data for this study were obtained from the Epic Cosmos database. There are restrictions to the availability of this data, which is used under license for this study. Data can be accessed with permission from the Epic Systems Corporation.

## References

[1] Kuipers EJ, Grady WM, Lieberman D, et al. Colorectal cancer. *Nat Rev Dis Primer*. 2015;1:15065. 10.1038/nrdp.2015.65. 10.1038/nrdp.2015.65 PMC4874655 27189416

[2] Khalil M, Munir MM, Woldesenbet S, et al. Association between historical redlining and access to high-volume hospitals among patients undergoing complex cancer surgery in California. *Ann Surg Oncol*. 2024;31(3):1477–87. 10.1245/s10434-023-14679-7. 38082168 10.1245/s10434-023-14679-7

[3] Hanna TP, King WD, Thibodeau S, et al. Mortality due to cancer treatment delay: systematic review and meta-analysis. *BMJ*. 2020;371:m4087. 10.1136/bmj.m4087. 33148535 10.1136/bmj.m4087 PMC7610021

[4] Khalil M, Munir MM, Endo Y, et al. Association of county-level food deserts and food swamps with hepatopancreatobiliary cancer outcomes. *J Gastrointest Surg Off J Soc Surg Aliment Tract*. 2023;27(12):2771–9. 10.1007/s11605-023-05879-3. 10.1007/s11605-023-05879-3 37940806

[5] Khan MMM, Munir MM, Woldesenbet S, et al. Association of COVID-19 Pandemic with colorectal cancer screening: impact of race/ethnicity and social vulnerability. *Ann Surg Oncol*. 2024;31(5):3222–32. 10.1245/s10434-024-15029-x. 38361094 10.1245/s10434-024-15029-x PMC10997707

[6] Seeman T, Epel E, Gruenewald T, Karlamangla A, McEwen BS. Socio-economic differentials in peripheral biology: cumulative allostatic load. *Ann N Y Acad Sci*. 2010;1186:223–39. 10.1111/j.1749-6632.2009.05341.x. 20201875 10.1111/j.1749-6632.2009.05341.x

[7] McEwen BS, Stellar E. Stress and the individual. Mechanisms leading to disease. *Arch Intern Med*. 1993;153(18):2093–101. 8379800

[8] McEwen BS. Allostasis and allostatic load: implications for neuropsychopharmacology. *Neuropsychopharmacol Off Publ Am Coll Neuropsychopharmacol*. 2000;22(2):108–24. 10.1016/S0893-133X(99)00129-3. 10.1016/S0893-133X(99)00129-3 10649824

[9] Goldstein DS, McEwen B. Allostasis, homeostats, and the nature of stress. *Stress Amst Neth*. 2002;5(1):55–8. 10.1080/102538902900012345. 10.1080/102538902900012345 12171767

[10] Obeng-Gyasi S, Elsaid MI, Lu Y, et al. Association of allostatic load with all-cause mortality in patients with breast cancer. *JAMA Netw Open*. 2023;6(5):e2313989. 10.1001/jamanetworkopen.2023.13989. 37200034 10.1001/jamanetworkopen.2023.13989 PMC10196875

[11] Carbone JT. Allostatic load and mental health: a latent class analysis of physiological dysregulation. *Stress Amst Neth*. 2021;24(4):394–403. 10.1080/10253890.2020.1813711. 10.1080/10253890.2020.1813711 32835575

[12] Stabellini N, Cullen J, Bittencourt MS, et al. Allostatic load/chronic stress and cardiovascular outcomes in patients diagnosed with breast, lung, or colorectal cancer. *J Am Heart Assoc Cardiovasc Cerebrovasc Dis*. 2024;13(14):e033295. 10.1161/JAHA.123.033295. 10.1161/JAHA.123.033295 PMC11292743 38979791

[13] Hyer JM, Tsilimigras DI, Diaz A, et al. High social vulnerability and “textbook outcomes” after cancer operation. *J Am Coll Surg*. 2021;232(4):351–9. 10.1016/j.jamcollsurg.2020.11.024. 33508426 10.1016/j.jamcollsurg.2020.11.024

[14] Tarabichi Y, Frees A, Honeywell S, et al. The Cosmos collaborative: a vendor-facilitated electronic health record data aggregation platform. *ACI Open*. 2021;5(1):e36–46. 10.1055/s-0041-1731004. 35071993 10.1055/s-0041-1731004 PMC8775787

[15] Hall ES, Melton GB, Payne PRO, Dorr DA, Vawdrey DK. How are leading research institutions engaging with data sharing tools and programs? *AMIA Annu Symp Proc*. 2024;2023:397–406. 38222386 PMC10785902

[16] Chen JC, Elsaid MI, Handley D, et al. Association between neighborhood opportunity, allostatic load, and all-cause mortality in patients with breast cancer. *J Clin Oncol Off J Am Soc Clin Oncol*. 2024;42(15):1788–98. 10.1200/JCO.23.00907. 10.1200/JCO.23.00907 PMC11095867 38364197

[17] Obeng-Gyasi S, Li Y, Carson WE, et al. Association of allostatic load with overall mortality among patients with metastatic non-small cell lung cancer. *JAMA Netw Open*. 2022;5(7):e2221626. 10.1001/jamanetworkopen.2022.21626. 35797043 10.1001/jamanetworkopen.2022.21626 PMC9264034

[18] CDC. Social vulnerability index. Place and health–geospatial research, analysis, and services program (GRASP). October 22, 2024. https://www.atsdr.cdc.gov/place-health/php/svi/index.html. Accessed 8 Jan 2025.

[19] Khalil M, Woldesenbet S, Munir MM, et al. Surgical outcomes and healthcare expenditures among patients with dementia undergoing major surgery. *World J Surg*. 2024;48(5):1075–83. 10.1002/wjs.12106. 38436547 10.1002/wjs.12106

[20] Khalil M, Woldesenbet S, Thammachack R, et al. Association of mental health assessment with postoperative outcomes following major surgery in older individuals. *Surgery*. 2024;180:109046. 10.1016/j.surg.2024.109046. 39740606 10.1016/j.surg.2024.109046

[21] Altaf A, Khalil M, Akabane M, et al. Up-front resection for hepatocellular carcinoma: assessing futility in the preoperative setting. *Eur J Surg Oncol J Eur Soc Surg Oncol Br Assoc Surg Oncol*. 2025;51(5):109594. 10.1016/j.ejso.2025.109594. 10.1016/j.ejso.2025.109594 39826445

[22] Krizanova O, Babula P, Pacak K. Stress, catecholaminergic system and cancer. *Stress Amst Neth*. 2016;19(4):419–28. 10.1080/10253890.2016.1203415. 10.1080/10253890.2016.1203415 27398826

[23] Morey JN, Boggero IA, Scott AB, Segerstrom SC. Current directions in stress and human immune function. *Curr Opin Psychol*. 2015;5:13–7. 10.1016/j.copsyc.2015.03.007. 26086030 10.1016/j.copsyc.2015.03.007 PMC4465119

[24] Ribeiro AI, Fraga S, Kelly-Irving M, et al. Neighbourhood socioeconomic deprivation and allostatic load: a multi-cohort study. *Sci Rep*. 2019;9(1):8790. 10.1038/s41598-019-45432-4. 31217447 10.1038/s41598-019-45432-4 PMC6584573

[25] Guidi J, Lucente M, Sonino N, Fava GA. Allostatic load and its impact on health: a systematic review. *Psychother Psychosom*. 2021;90(1):11–27. 10.1159/000510696. 32799204 10.1159/000510696

[26] Juster RP, Russell JJ, Almeida D, Picard M. Allostatic load and comorbidities: a mitochondrial, epigenetic, and evolutionary perspective. *Dev Psychopathol*. 2016;28(4(pt1)):1117–46. 10.1017/S0954579416000730. 27739386 10.1017/S0954579416000730

[27] Pfaltz MC, Schnyder U. Allostatic load and allostatic overload: preventive and clinical implications. *Psychother Psychosom*. 2023;92(5):279–82. 10.1159/000534340. 37931612 10.1159/000534340 PMC10716872

[28] Glasheen WP, Cordier T, Gumpina R, Haugh G, Davis J, Renda A. Charlson comorbidity index: ICD-9 update and ICD-10 translation. *Am Health Drug Benefits*. 2019;12(4):188–97. 31428236 PMC6684052

[29] Liu Y, Cohen ME, Hall BL, Ko CY, Bilimoria KY. Evaluation and enhancement of calibration in the American college of surgeons NSQIP surgical risk calculator. *J Am Coll Surg*. 2016;223(2):231–9. 10.1016/j.jamcollsurg.2016.03.040. 27212006 10.1016/j.jamcollsurg.2016.03.040

