# Supplementary material for: The Hidden Burden: Impact of Allostatic Load on Colorectal Cancer Surgery Outcomes
Source: Ann Surg Oncol. 2025 Jun 21;32(10):7723–30. doi: 10.1245/s10434-025-17711-0 (PMC12454506; doi:10.1245/s10434-025-17711-0)
Supplement: Supplementary file 1 — Supplementary file1 (DOCX 17 KB) [file 10434_2025_17711_MOESM1_ESM.docx]

**Supplementary Table 1: Risk factors for high allostatic load.**

| **Factors** | **Odds ratio** | **95% CI** | **P value** |
| --- | --- | --- | --- |
| Age (1 year increase) | 1.01 | 1.02 – 1.02 | <0.001 |
| Marital status |  |  | <0.001 |
| Single | Ref |  |  |
| Married | 0.84 | 0.78 – 0.90 |  |
| Race |  |  |  |
| White | Ref |  |  |
| Black | 1.73 | 1.58 – 1.89 | 0.000 |
| Asian | 0.83 | 0.67 – 1.03 | 0.087 |
| Other | 1.15 | 0.96 – 1.37 | 0.135 |
| Sex |  |  |  |
| Female |  |  | <0.001 |
| Male | 1.67 | 1.56 – 1.79 |  |
| Charlson Comorbidity Index |  |  | <0.001 |
| ≤2 | Ref |  |  |
| >2 | 2.93 | 2.61 – 3.29 |  |
| Social vulnerability index |  |  |  |
| Low | Ref |  | <0.001 |
| Medium | 1.10 | 1.01 – 1.20 | 0.022 |
| High | 1.17 | 1.07 – 1.28 | <0.001 |
| Residential area |  |  | 0.023 |
| Metropolitan | Ref |  |  |
| Non-metrometropolitan | 1.10 | 1.01 – 1.20 |  |

**Supplementary Table 2: Odds of complications among patients with high allostatic load, stratified by clinicodemographic factors.**

| **Factors** | **Odds of complications** | **95% CI** | **p value** |
| --- | --- | --- | --- |
| CCI |  |  |  |
| ≤2 | 1.63 | 1.30 – 2.05 | <0.001 |
| >2 | 1.46 | 1.36 – 1.57 | <0.001 |
| Cancer site |  |  |  |
| Colon | 1.48 | 1.38 – 1.60 | <0.001 |
| Rectum | 1.44 | 1.21 – 1.72 | <0.001 |
| Surgical approach |  |  |  |
| Open | 1.50 | 1.38 – 1.64 | <0.001 |
| MIS | 1.44 | 1.30 – 1.60 | <0.001 |
| Social Vulnerability Index |  |  |  |
| Low | 1.54 | 1.36 – 1.75 | <0.001 |
| Medium | 1.42 | 1.27 – 1.60 | <0.001 |
| High | 1.48 | 1.33 – 1.65 | <0.001 |
